# Machine Learning-Based Prediction of Early Patient-Controlled Analgesia Discontinuation After Total Knee Arthroplasty: A Retrospective Cohort Study

**DOI:** 10.3390/jcm15114282

**Published:** 2026-06-01

**Authors:** Sang Gyu Kwak, Jae Bum Kwon, Won Kee Choi

**Affiliations:** 1 Department of Medical Statistics, Daegu Catholic University School of Medicine, Daegu 42472, Republic of Korea; sgkwak@cu.ac.kr; 2 Department of Orthopaedic Surgery, Daegu Catholic University School of Medicine, Daegu 42472, Republic of Korea; jbkwon@cu.ac.kr

**Keywords:** patient-controlled analgesia, total knee arthroplasty, postoperative pain management, machine learning

## Abstract

**Background:** Patient-controlled analgesia (PCA) is widely used for postoperative pain control after total knee arthroplasty (TKA). Although postoperative nausea and vomiting (PONV) has been extensively studied, early discontinuation of PCA—representing failure to sustain an opioid-based analgesic strategy—has not been adequately investigated as a distinct, decision-relevant outcome. **Methods:** We conducted a single-center retrospective observational study of 1188 patients undergoing primary TKA who received PCA for postoperative pain management. The primary aim was to develop interpretable machine learning models for predicting early PCA discontinuation using routinely available perioperative variables. A secondary aim was to evaluate the incremental predictive value of hierarchical feature sets reflecting progressively available clinical information. **Results:** Early PCA discontinuation occurred in approximately 10% of patients, reflecting a relatively low-frequency clinical event associated with class imbalance. Female sex and PONV-related susceptibility factors, including prior nausea/vomiting intolerance, were more common among patients with early PCA discontinuation. The random forest Step 3 model demonstrated acceptable discriminative performance (AUC: 0.77; PR-AUC: 0.38), good calibration accuracy (Brier score: 0.065), and favorable clinical utility on decision curve analysis. Explainable analyses showed that patient-level susceptibility factors—such as prior intolerance to nausea or vomiting and baseline clinical characteristics—contributed more strongly to early PCA discontinuation than perioperative management or procedural variables. **Conclusions:** Early PCA discontinuation after TKA represents a distinct, decision-based clinical endpoint that is not captured by symptom-focused outcomes such as PONV. Interpretable machine learning models may help identify patients at increased risk of early PCA discontinuation after TKA, which may support informed counseling and proactive planning of individualized postoperative pain management strategies. However, external validation is required before routine clinical implementation.

## 1. Introduction

Total knee arthroplasty (TKA) is a widely performed surgical procedure for end-stage knee osteoarthritis and is associated with substantial improvements in pain and function. Nevertheless, the early postoperative period is frequently characterized by severe acute pain, which remains a primary concern for patients and a key determinant of patient satisfaction, early mobilization, and overall acceptance of surgical outcomes [1,2]. Postoperative pain intensity after TKA is commonly evaluated using patient-reported measures such as the numeric rating scale (NRS) and visual analog scale (VAS), with the NRS frequently preferred because of its simplicity and ease of patient interpretation. However, although these scales quantify symptom severity, they do not necessarily reflect the feasibility of maintaining a postoperative analgesic strategy over time.

Postoperative pain management after TKA is delivered through multimodal analgesic pathways that integrate non-opioid medications, local infiltration analgesia, regional nerve blocks, and systemic opioids. Contemporary perioperative care has increasingly emphasized opioid-sparing and multimodal analgesic strategies within Enhanced Recovery After Surgery (ERAS) pathways to reduce opioid-related adverse effects and facilitate early functional recovery after TKA. Within ERAS protocols, minimizing opioid-related complications and maintaining tolerable postoperative analgesia are considered important components of optimized recovery pathways after TKA. Accordingly, alternative postoperative pain management strategies, including opioid-sparing multimodal analgesia, regional nerve blocks, and enhanced local infiltration analgesia, may be considered in patients anticipated to have difficulty tolerating opioid-based PCA [1,2,3]. Within these pathways, opioid-based systemic analgesia administered via patient-controlled analgesia (PCA) remains commonly used during the early postoperative period in many institutions, reflecting the intensity of postoperative pain and the need for flexible, patient-responsive analgesia. Although PCA is not universally applied, it continues to represent a routine component of postoperative pain control for a substantial proportion of patients undergoing TKA [1,3,4].

A considerable body of perioperative research has focused on predicting the occurrence of postoperative nausea and vomiting (PONV), given its high incidence and impact on patient comfort. These studies have identified patient susceptibility factors associated with postoperative emetic symptoms and contributed to improved prophylactic strategies [5,6,7].

In contrast, early discontinuation of PCA represents a fundamentally different clinical phenomenon. Rather than reflecting the mere occurrence of postoperative symptoms, PCA discontinuation indicates an inability to sustain an opioid-based analgesic strategy during the critical early recovery phase. This outcome is commonly associated with escalation of rescue analgesia, disruption of postoperative care pathways, and reduced patient satisfaction, underscoring that early PCA discontinuation is a clinically meaningful marker of analgesic strategy failure rather than a surrogate for postoperative nausea or vomiting alone [8].

Despite its clinical relevance, early PCA discontinuation has received limited attention as a primary endpoint in perioperative research. Unlike isolated symptom scores or rescue opioid consumption, PCA discontinuation directly reflects the inability to sustain the intended analgesic strategy during the critical early postoperative period and therefore represents a pragmatic treatment-level endpoint relevant to real-world perioperative decision-making [5,6,7,8]. Most existing studies have continued to focus on symptom prediction, particularly PONV, rather than evaluating the feasibility of maintaining analgesic strategies themselves. Consequently, factors that distinguish patients who can tolerate PCA from those who are unable to sustain its use remain poorly characterized, leaving clinicians to respond reactively after pain control has already failed.

Advances in machine learning (ML) offer an opportunity to address this gap by enabling individualized risk prediction using routinely available perioperative data and by modeling complex, nonlinear relationships among patient characteristics and clinical variables. When combined with interpretable modeling approaches, ML-based predictions can support transparent medical decision-making, facilitating more accurate preoperative counseling and proactive planning of alternative or opioid-sparing analgesic strategies in patients at high risk of PCA intolerance [9,10,11].

Therefore, the objective of this study was to develop and evaluate an interpretable machine learning-based model to predict early discontinuation of patient-controlled analgesia within 24 h after total knee arthroplasty using routinely available perioperative data. By conceptualizing early PCA discontinuation as a marker of analgesic strategy failure rather than isolated symptom occurrence, this study aims to provide clinically interpretable risk stratification support to guide individualized postoperative pain management and shared medical decision-making in patients undergoing TKA.

## 2. Materials and Methods

### 2.1. Study Design and Population

This study was a single-center, retrospective observational study conducted using electronic medical record (EMR) data from adult patients who underwent total knee arthroplasty (TKA) at Daegu Catholic University Medical Center. All consecutive adult patients who received primary TKA under either general or regional (spinal) anesthesia between 1 December 2015 and 30 November 2025 were screened for eligibility.

Patients were eligible for inclusion if postoperative pain control with patient-controlled analgesia (PCA) was initiated immediately after surgery and the status of PCA maintenance or discontinuation within the first 24 h after surgery could be clearly ascertained. Only unilateral total knee arthroplasty procedures were included, whereas bilateral procedures were excluded to ensure independence of observations. Patients were excluded if PCA was not applied postoperatively, if PCA discontinuation status within 24 h could not be reliably determined, or if major intraoperative or immediate postoperative complications, death, transfer to the intensive care unit, or reoperation within 24 h after surgery precluded evaluation of PCA use. Patients with substantial missing data in the outcome variable or key perioperative predictors required for model development were excluded from the analysis (*n* = 14, 1.2%). Because the overall proportion of missing data was low, no imputation methods were applied, and complete-case analysis was performed.

After application of the inclusion and exclusion criteria, a total of 1188 patients were included in the final analytic cohort and stratified according to whether PCA was maintained or discontinued within 24 h after surgery. A summary of the inclusion and exclusion criteria is additionally provided in Supplementary Table S4.

The study protocol was reviewed and approved by the Institutional Review Board of Daegu Catholic University Medical Center (IRB No. DCUMC 2026-01-044).

The requirement for informed consent was waived owing to the retrospective nature of the study and the use of de-identified patient data. All study procedures were conducted in accordance with the Declaration of Helsinki and relevant institutional guidelines.

### 2.2. Data Collection and Variables

Clinical data were retrospectively extracted from the electronic medical record (EMR) system, including anesthesia records, operative reports, postoperative nursing notes, medication administration records, and preoperative medical histories. Data extraction was performed using a standardized case report form, and all collected variables were anonymized prior to analysis. To ensure clinical applicability and avoid information leakage, only variables available before or during surgery were considered as candidate predictors. Postoperative variables, including those obtained in the post-anesthesia care unit or after surgery completion, were not included as predictors.

Baseline demographic and clinical variables included age, sex, body mass index, smoking status, and major comorbidities. Comorbid conditions were identified based on preoperative diagnoses documented in the EMR and included hypertension, diabetes mellitus, cardiovascular disease, cerebrovascular disease, chronic lung disease, and chronic kidney disease. Variables related to susceptibility to postoperative nausea and vomiting (PONV) were collected from preoperative assessments and medical histories. These included a documented history of PONV or motion sickness and a history of analgesic-related adverse drug reactions accompanied by nausea and/or vomiting. Analgesic-related ADR with NV was defined as a preoperative history of nausea and/or vomiting associated with analgesic exposure documented before surgery. Perioperative variables reflected intraoperative anesthesia and analgesia management and included anesthesia type (general or regional), use of local infiltration analgesia, and intraoperative administration of dexamethasone. These variables were selected because they are routinely available at the end of surgery and may influence early postoperative tolerance to PCA [12,13,14,15,16]. During the study period, intravenous opioid-based PCA was routinely administered as part of the institutional postoperative pain management pathway after TKA. PCA regimens were prescribed according to institutional practice patterns and generally consisted of opioid-based formulations with adjunctive non-opioid analgesic strategies as clinically indicated. Decisions regarding continuation or discontinuation of PCA were made based on patient tolerance, adverse effects, and clinical judgment during the early postoperative period. Perioperative antiemetic prophylaxis, including dexamethasone administration, was performed according to institutional anesthesia practice and individualized clinical risk assessment rather than a strictly uniform protocol. The institutional postoperative pain management pathway additionally incorporated multimodal analgesic strategies, including local infiltration analgesia and rescue analgesic administration as clinically indicated during postoperative recovery. Although perioperative management generally followed institutional clinical practice patterns, complete uniformity across surgeons and anesthesia providers throughout the extended retrospective study period could not be guaranteed.

The primary outcome was early discontinuation of patient-controlled analgesia (PCA), defined as termination, removal, or cessation of PCA use for any reason within 24 h after the end of surgery. PCA discontinuation included cases in which PCA was stopped due to adverse drug reactions (e.g., nausea, vomiting, dizziness, pruritus, or excessive sedation), patient discomfort or refusal, or clinical judgment by the attending physician or anesthesiologist. Temporary interruption of PCA followed by re-initiation within 24 h was not considered discontinuation.

### 2.3. Construction of Three Hierarchical Feature Sets

To evaluate the incremental predictive value of additional clinical information available at different stages of perioperative care, three hierarchical feature sets were constructed a priori. Each feature set reflected the progressive availability of patient information from baseline characteristics to perioperative management variables and was designed to align with real-world clinical decision-making at the end of surgery.

The first feature set (Step 1) included baseline demographic characteristics and pre-existing comorbidities routinely available before surgery. Variables in this feature set comprised age, sex, body mass index, smoking status, hypertension, diabetes mellitus, cardiovascular disease, cerebrovascular disease, chronic lung disease, and chronic kidney disease. This feature set represented a minimal model based solely on patient-related characteristics. The second feature set (Step 2) expanded upon Step 1 by incorporating variables related to individual susceptibility to postoperative nausea and vomiting. These variables included a documented history of postoperative nausea and vomiting or motion sickness and a preoperative history of analgesic-related adverse drug reactions accompanied by nausea and/or vomiting. The third feature set (Step 3) further extended Step 2 by adding perioperative anesthesia and analgesia management variables available at the completion of surgery. These included anesthesia type (general versus regional), use of local infiltration analgesia, and intraoperative administration of dexamethasone.

### 2.4. Model Development and Evaluation

Three machine learning algorithms were used to develop prediction models for early discontinuation of patient-controlled analgesia (PCA): logistic regression (LR), random forest (RF), and extreme gradient boosting (XGBoost) [12,17]. Logistic regression with L2 regularization was included as a baseline model to reduce overfitting, while tree-based models were employed to capture nonlinear relationships and interactions among predictors. For each model, hyperparameters were optimized using stratified five-fold cross-validation performed exclusively within the training set. The independent test set was not used during model training, feature selection, or hyperparameter optimization procedures in order to minimize information leakage and optimistic bias. Detailed hyperparameter search spaces and optimization procedures are provided in the Supplementary Materials.

The full dataset was randomly split into a training set (80%) and an independent test set (20%) using stratified sampling to preserve the proportion of PCA discontinuation events. Model development, including feature set evaluation and hyperparameter optimization, was conducted exclusively within the training set, and the same data split was used consistently across all models and feature sets to ensure fair comparison.

Given the relatively low event rate of PCA discontinuation and the resulting class imbalance, the imbalance was addressed during model training by applying class weights inversely proportional to class frequencies within the training set for logistic regression, random forest, and XGBoost models without modifying the original data distribution. This approach was selected to preserve the observed clinical event prevalence and reduce potential distortion associated with synthetic oversampling techniques. Alternative balancing approaches, including the synthetic minority oversampling technique (SMOTE) and undersampling, were not evaluated because the present study prioritized preservation of the original clinical event distribution and real-world prevalence. This approach allowed greater emphasis on identifying PCA discontinuation events without altering the underlying data distribution [18]. PR-AUC was prioritized as a key performance metric because PCA discontinuation represented a relatively low-frequency clinical event, for which precision-recall analysis provides a more informative evaluation than ROC-based metrics alone.

Model performance was evaluated in terms of discrimination using the area under the receiver operating characteristic curve (AUC) and the area under the precision–recall curve (PR-AUC). Cross-validated performance was summarized as the mean and standard deviation of AUC and PR-AUC across the five validation folds in the training set. Final model performance was assessed on the independent test set using the same metrics. Model calibration and overall prediction accuracy were additionally assessed using calibration plots and the Brier score on the independent test set. Lower Brier scores indicate better agreement between predicted probabilities and observed outcomes. Clinical utility was further evaluated using decision curve analysis (DCA), which estimates the net benefit of model-guided decision-making across a range of threshold probabilities. Ninety-five percent confidence intervals for test-set AUC and PR-AUC values were estimated using bootstrap resampling with 1000 iterations.

To enhance interpretability, feature importance was assessed for each model. For logistic regression, feature importance was defined as the absolute values of standardized regression coefficients. For random forest, feature importance was quantified based on the frequency of feature usage across tree splits, and for XGBoost, gain-based feature importance was calculated. Model-agnostic explainability was further evaluated using SHapley Additive exPlanations (SHAP). SHAP analyses were performed using the independent test set after completion of final model development. Mean absolute SHAP values were used to summarize the overall contribution of each feature to model predictions, and SHAP summary plots were generated to visualize both the magnitude and direction of association between predictors and the risk of early PCA discontinuation [19].

### 2.5. Statistical Analysis

Descriptive statistics were used to summarize baseline patient characteristics and perioperative variables. Continuous variables are presented as mean ± standard deviation, and categorical variables are presented as counts and percentages. Comparisons between patients who maintained PCA and those who discontinued PCA within 24 h were performed using the independent two-sample *t*-test for continuous variables and the chi-square test or Fisher’s exact test for categorical variables, as appropriate. All statistical analyses and machine learning model development procedures were performed using Python (version 3.11), with the use of the scikit-learn, XGBoost, and SHAP libraries. All statistical tests were two-sided, and a *p*-value < 0.05 was considered statistically significant. Statistical significance testing was applied only to descriptive group comparisons; machine learning model performance was evaluated using discrimination, calibration, and decision curve analyses without formal hypothesis testing. This study was conducted and reported in accordance with the TRIPOD + AI statement for clinical prediction model studies using machine learning methods [20].

## 3. Results

**Figure 1:**
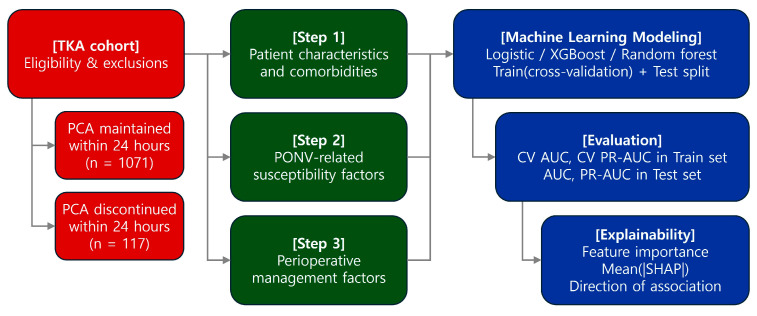
Study flow and machine learning pipeline for predicting patient-controlled analgesia discontinuation within 24 h after total knee arthroplasty. Step 1 includes baseline characteristics and comorbidities; Step 2 additionally incorporates PONV-related susceptibility factors; and Step 3 further includes perioperative management variables. AUC, area under the receiver operating characteristic curve; PCA, patient-controlled analgesia; PR-AUC, area under the precision–recall curve; TKA, total knee arthroplasty.

Figure 1 illustrates the overall study flow, including patient selection, construction of hierarchical feature sets, data splitting, model development, and evaluation procedures.

### 3.1. Study Population and Baseline Characteristics

A total of 1188 adult patients who underwent unilateral total knee arthroplasty and received postoperative patient-controlled analgesia (PCA) were included in the final analysis. Among these patients, PCA was discontinued within 24 h after surgery in 117 patients (9.9%), whereas 1071 patients (90.1%) maintained PCA beyond 24 h. Baseline patient characteristics and comorbidities according to PCA discontinuation status are summarized in Table 1. Patients who discontinued PCA within 24 h were significantly more likely to be female than those who maintained PCA (90.6% vs. 79.7%, *p* = 0.007). Current smoking was less frequent in the PCA discontinuation group compared with the PCA maintenance group (4.3% vs. 11.4%, *p* = 0.027). There were no significant differences between the two groups in age, body mass index, or the prevalence of major comorbidities, including hypertension, diabetes mellitus, cardiovascular disease, cerebrovascular disease, chronic lung disease, and chronic kidney disease.

PONV-related susceptibility factors and perioperative management variables are presented in Table 2. The history of postoperative nausea and vomiting or motion sickness was significantly more common among patients who discontinued PCA within 24 h compared with those who maintained PCA (41.0% vs. 17.8%, *p* < 0.001). Similarly, a preoperative history of analgesic-related adverse drug reactions accompanied by nausea and/or vomiting was more frequently observed in the PCA discontinuation group (34.2% vs. 13.2%, *p* < 0.001). In contrast, no significant differences were observed between groups with respect to anesthesia type, use of local infiltration analgesia, or intraoperative dexamethasone administration.

### 3.2. Model Performance Across Hierarchical Feature Sets

Predictive performance of the machine learning models across the three hierarchical feature sets is summarized in Table 3. In Step 1, which included only baseline demographic characteristics and comorbidities, all models demonstrated limited discriminative performance, with test AUC values ranging from 0.546 to 0.664. Addition of PONV-related susceptibility factors in Step 2 resulted in a substantial improvement in both cross-validated and test-set performance across all models. Further inclusion of perioperative management variables in Step 3 resulted in minimal additional improvement in predictive performance, although these variables were retained to reflect clinically available perioperative information at the completion of surgery.

**Figure 2:**
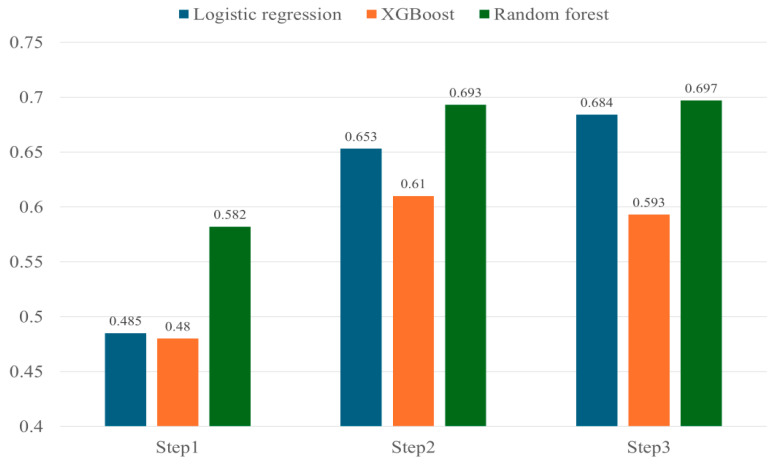
Comparison of predictive performance across machine learning models and stepwise feature sets for predicting PCA discontinuation within 24 h. Step 1 includes baseline characteristics and comorbidities; Step 2 additionally incorporates PONV-related susceptibility factors; and Step 3 further includes perioperative management variables.

Across all feature sets, the random forest model consistently demonstrated the highest discriminative performance among the evaluated models. In Step 3, the random forest model achieved a test AUC of 0.773 (95% CI, 0.600–0.941) and a test PR-AUC of 0.378 (95% CI, 0.273–0.549). A comparison of model performance across feature sets and algorithms is illustrated in Figure 2, demonstrating improved discriminative performance from Step 1 to Step 2, with minimal further improvement in Step 3. Additional threshold-dependent classification metrics, including sensitivity, specificity, positive predictive value, and negative predictive value, are provided in Supplementary Table S5 and should be interpreted as exploratory given the absence of a predefined clinical probability threshold.

### 3.3. Receiver Operating Characteristic and Precision–Recall Analyses

**Figure 3:**
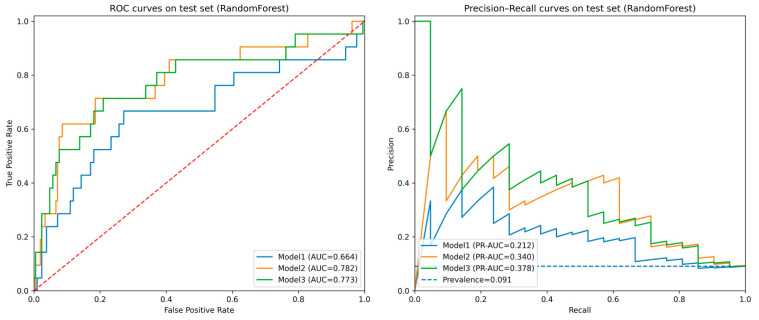
Receiver operating characteristic (ROC) and precision–recall (PR) curves of the random forest model across stepwise feature sets. ROC (**left**) and PR (**right**) curves are shown for the random forest model evaluated on the independent test set across the three hierarchical feature sets. The diagonal dashed line in the ROC panel represents chance-level discrimination, and the horizontal dashed line in the PR panel indicates the event prevalence in the test set. AUC, area under the receiver operating characteristic curve; PR-AUC, area under the precision–recall curve; ROC, receiver operating characteristic.

Receiver operating characteristic (ROC) and precision–recall (PR) curves for the random forest model evaluated on the independent test set are shown in Figure 3. Substantial improvements in both ROC and PR curves were observed from Step 1 to Step 2, while the addition of perioperative management variables in Step 3 resulted in minimal further change, indicating enhanced discrimination and improved identification of PCA discontinuation events with the inclusion of PONV-related susceptibility and perioperative management variables.

Corresponding ROC and PR curves for the logistic regression and XGBoost models are provided in Supplementary Figures S2 and S3, respectively. These models demonstrated similar stepwise trends across feature sets, although their overall performance was inferior to that of the random forest model.

### 3.4. Calibration and Decision Curve Analyses

**Figure 4:**
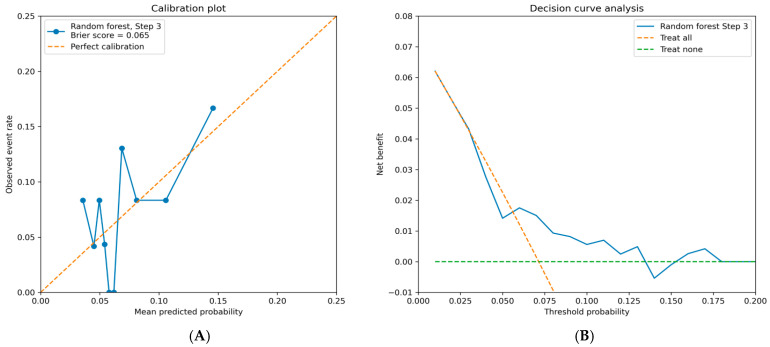
Calibration and clinical utility of the Step 3 random forest model. (**A**) Calibration plot comparing predicted and observed probabilities of PCA discontinuation within 24 h. The dashed diagonal line represents perfect calibration. (**B**) Decision curve analysis showing net benefit across threshold probabilities compared with treat-all and treat-none strategies.

Calibration and decision curve analyses for the Step 3 random forest model are presented in Figure 4. Calibration assessment was primarily based on calibration plots and the Brier score. Calibration plots demonstrated acceptable agreement between predicted and observed probabilities of PCA discontinuation within 24 h. The random forest Step 3 model showed a Brier score of 0.065, indicating acceptable overall prediction accuracy and calibration. Calibration intercept and slope were not additionally evaluated. Decision curve analysis demonstrated that the model provided greater net benefit than default treat-all or treat-none strategies across threshold probabilities of approximately 5% to 15%, supporting its potential utility for individualized perioperative risk stratification.

### 3.5. Feature Importance and Model Explainability

Feature importance analyses reveal consistent patterns across models and feature sets. Detailed feature importance metrics, mean absolute SHAP values, and directions of association for each model are presented in Supplementary Tables S1–S3.

In the Step 3 random forest model, PONV-related susceptibility factors—including a history of postoperative nausea and vomiting or motion sickness and a preoperative history of analgesic-related adverse drug reactions with nausea and/or vomiting—emerged as the most influential predictors of early PCA discontinuation. Baseline patient characteristics such as body mass index, age, and sex contributed modestly to model predictions, whereas most comorbidities and perioperative management variables showed relatively smaller contributions. A SHAP summary plot for the Step 3 random forest model is shown in Supplementary Figure S1, illustrating both the magnitude and direction of association between individual predictors and the risk of PCA discontinuation within 24 h.

## 4. Discussion

The present study demonstrates that early discontinuation of patient-controlled analgesia (PCA) after total knee arthroplasty (TKA), although observed in a minority of patients, represents a clinically meaningful and predictable event. In practical terms, these findings indicate that failure to maintain PCA is driven more by patient tolerance than by perioperative technique. By focusing on the feasibility of sustaining an opioid-based analgesic strategy rather than on isolated postoperative symptoms, this study positions PCA discontinuation as a decision-relevant endpoint with direct implications for postoperative pain management.

An important implication of this finding is that early PCA discontinuation should be clearly distinguished from symptom-based postoperative outcomes such as postoperative nausea and vomiting (PONV). In routine clinical practice, transient adverse symptoms are often managed without altering the overall analgesic plan. By contrast, PCA discontinuation reflects cumulative intolerance that ultimately leads to a treatment-level decision to abandon opioid-based analgesia. This distinction explains why PCA discontinuation should not be interpreted as a surrogate marker of PONV and why predictors of these two outcomes are not expected to fully overlap [5,21].

The present results show partial concordance with prior PONV-focused studies in that patient-level susceptibility factors contributed substantially to risk prediction. This consistency suggests that intrinsic vulnerability to opioid-related adverse effects remains central to postoperative intolerance [6,7,8]. However, the present study extends previous work by demonstrating that such susceptibility factors are more strongly associated with the inability to sustain analgesic therapy than with symptom occurrence alone, underscoring the importance of examining treatment feasibility rather than symptom incidence.

Baseline patient characteristics were included to capture physiological factors that may modulate opioid sensitivity during the early postoperative period. The prominence of these variables in explainable analyses indicates that PCA discontinuation reflects cumulative pharmacologic intolerance rather than isolated perioperative exposure [22]. In contrast, perioperative management and procedural variables demonstrated relatively modest contributions. This finding suggests that standardized perioperative pathways may limit the influence of technique-related factors once a baseline level of care is achieved [2,4,23]. In addition, evolving perioperative approaches, including remimazolam-based anesthesia, robotic-assisted total knee arthroplasty (RA-TKA), and alternative postoperative analgesic strategies such as subcutaneous patient-controlled analgesia and opioid-sparing multimodal regimens incorporating agents such as ketorolac or nefopam, may further influence postoperative pain experience and tolerance to opioid-based analgesic strategies [24,25,26,27]. Such approaches may be particularly relevant for patients identified as being at increased risk of early PCA discontinuation. Continuous femoral nerve blockade has also been compared with conventional intravenous patient-controlled analgesia after total knee arthroplasty and may provide effective postoperative analgesia while reducing reliance on systemic opioids, although potential effects on motor function and early mobilization should be considered [28,29]. However, because these approaches were not consistently applied during the present study period, their effects could not be specifically evaluated in the current analysis. Moreover, because PCA discontinuation occurs after initial symptom management, it is inherently shaped by patient response over time rather than by immediate intraoperative factors.

Although early PCA discontinuation occurred in a relatively small proportion of patients, this does not imply limited clinical importance. Failure to maintain PCA is frequently associated with inadequate pain control, increased reliance on rescue analgesics, and disruption of established postoperative care pathways [8]. Thus, a low event rate should be interpreted not as triviality, but as an indicator of a high-impact event affecting a vulnerable subset of patients.

The quantitative performance of the prediction model further informs its intended clinical role. The observed discriminative performance was moderate and suggests potential utility for perioperative risk stratification rather than definitive prediction in routine clinical practice. In addition, calibration analysis demonstrated good agreement between predicted and observed event probabilities, while decision curve analysis suggested potential clinical utility across clinically relevant threshold probabilities. These findings suggest potential applicability of the model for perioperative risk stratification rather than a purely statistical prediction model. Moreover, the relatively low Brier score and favorable calibration profile suggest that predicted probabilities were reasonably aligned with observed event rates, supporting the reliability of individualized risk estimation. Because the Brier score reflects overall probability error and calibration rather than discrimination alone, relatively similar Brier scores were observed across logistic regression models despite substantial differences in AUC values, particularly in the setting of low event incidence. Notably, logistic regression models demonstrated generally comparable performance across several feature sets despite their substantially simpler structure. This finding suggests that parsimonious and more interpretable statistical models may still provide clinically meaningful risk stratification while offering advantages in transparency and implementation feasibility compared with more complex machine learning approaches.

Importantly, precision–recall metrics should be interpreted in the context of the relatively low incidence of early PCA discontinuation. Although PR-AUC values remained modest, this finding is expected in low-prevalence prediction settings with substantial class imbalance. Although the event rate was relatively low, the study included 117 PCA discontinuation events, and several methodological strategies—including stratified data splitting, cross-validation, regularization, and class-weighting approaches—were applied to reduce the risk of model instability and overfitting. Within this setting, the model may help identify patients at comparatively higher risk, rather than as a binary decision rule. Explainable analyses consistently demonstrated that patient-level susceptibility factors contributed more strongly to predicted risk than procedural variables, reinforcing the conceptualization of PCA discontinuation as a tolerance-driven outcome.

From a clinical decision-making perspective, these findings suggest that risk prediction should be used to inform, rather than restrict, analgesic choices. Identification of patients at increased risk of early PCA discontinuation may support more accurate preoperative counseling, alignment of expectations regarding postoperative pain, and proactive consideration of alternative or opioid-sparing strategies such as enhanced local infiltration analgesia or regional nerve blocks [13,14]. In this context, interpretability is essential, as transparent risk estimation facilitates meaningful communication between clinicians and patients.

Several aspects distinguish the present study from existing perioperative research. Whereas most prior studies have focused on predicting postoperative symptoms such as PONV, this study addresses whether a primary analgesic strategy can be sustained. By examining PCA discontinuation as a treatment-level endpoint, the present work provides clinically actionable insights that extend beyond symptom prediction and align more closely with real-world perioperative decision-making.

Several limitations warrant consideration. This retrospective, single-institution study may have limited generalizability to settings with different analgesic protocols or patient populations. In addition, some potentially relevant perioperative variables, including ASA physical status classification and chronic steroid use, were not consistently available across the retrospective dataset and therefore could not be evaluated. PCA discontinuation is inherently influenced by clinical judgment, which may introduce variability; however, this characteristic also reflects routine clinical practice rather than protocol-driven abstraction. In addition, perioperative management practices may have varied across clinicians and over time within the institution, potentially introducing institutional practice bias. Changes in surgical techniques, anesthesia approaches, postoperative analgesic strategies, and perioperative care pathways during the extended 2015–2025 study period may also have contributed to temporal heterogeneity and unmeasured confounding. Although PCA discontinuation may occur for heterogeneous clinical reasons, including nausea, vomiting, dizziness, pruritus, sedation, and patient or physician preference, the present study intentionally conceptualized discontinuation as a pragmatic treatment-level endpoint reflecting failure to sustain opioid-based analgesia in real-world clinical practice. Patients with a documented history of severe opioid intolerance were less likely to receive PCA, potentially leading to under-representation of the highest-risk individuals and attenuation of observed associations. The analysis focused on early postoperative events, and later discontinuation was not captured. In addition, patient-reported outcome measures, including postoperative pain severity, patient satisfaction, and recovery experience, were not available and therefore could not be incorporated into the present analysis. Because the present model was developed and evaluated using retrospective single-center data without external validation and demonstrated only moderate predictive performance, its generalizability and applicability across institutions with different patient populations, anesthesia practices, PCA protocols, multimodal analgesic strategies, and perioperative care pathways remain uncertain. Future prospective studies incorporating real-time risk stratification may further support the clinical integration of this prediction framework.

Future implementation studies may additionally explore the integration of prediction models into electronic medical record systems to enable automated perioperative risk estimation using routinely collected clinical variables. Prospective multicenter validation across institutions with different analgesic pathways and perioperative management strategies will be necessary to evaluate generalizability and clinical robustness. In addition, practical challenges related to workflow integration, clinician acceptance, alert fatigue, and interpretation of model-generated risk estimates should be carefully considered before routine clinical deployment. Future studies incorporating patient-reported outcome measures (PROMs), including postoperative pain severity, treatment satisfaction, functional recovery, and overall recovery experience, may further enhance evaluation of individualized postoperative analgesic strategies beyond PCA discontinuation alone. More broadly, emerging evidence suggests that pain perception and treatment response may be influenced by multidimensional physiological and behavioral factors beyond conventional perioperative variables, highlighting the complexity of individualized pain assessment and management [30,31].

## 5. Conclusions

Early discontinuation of patient-controlled analgesia after total knee arthroplasty represents a clinically meaningful outcome reflecting failure to sustain opioid-based analgesia rather than isolated postoperative symptoms. Using interpretable machine learning, this study suggests that early PCA discontinuation is more strongly associated with patient-level susceptibility factors and may be identifiable before surgery. Nevertheless, external validation will be necessary before routine clinical implementation. These findings support the role of preoperative risk stratification in guiding individualized postoperative pain management decisions.

## Figures and Tables

**Table 1:** Baseline patient characteristics and comorbidities according to PCA discontinuation within 24 h.

Variable	PCA Maintained Within 24 h (*n* = 1071)	PCA Discontinued Within 24 h (*n* = 117)	*p*-Value
Age (years)	74.35 ± 7.27	74.66 ± 7.72	0.679
BMI (kg/m^2^)	26.03 ± 4.34	27.05 ± 5.58	0.058
Sex, female	854 (79.7)	106 (90.6)	0.007
Smoking, yes	122 (11.4)	5 (4.3)	0.027
HTN, yes	630 (58.8)	63 (53.8)	0.348
DM, yes	275 (25.7)	28 (23.9)	0.765
CVD, yes	120 (11.2)	17 (14.5)	0.359
CeVD, yes	74 (6.9)	10 (8.5)	0.641
CLD, yes	13 (1.2)	3 (2.6)	0.435
CKD, yes	32 (3.0)	2 (1.7)	0.620

Values were presented by frequency (percentage) or mean ± standard deviation; *p*-values were calculated by chi-square test for categorical variables and independent two-sample *t*-test for continuous variables, as appropriate; BMI, body mass index; CeVD, cerebrovascular disease; CKD, chronic kidney disease; CLD, chronic lung disease; CVD, cardiovascular disease; DM, diabetes mellitus; HTN, hypertension; PCA, patient-controlled analgesia.

**Table 2:** PONV-related susceptibility and perioperative management factors by PCA discontinuation within 24 h.

Variable	PCA Maintained Within 24 h (*n* = 1071)	PCA Discontinued Within 24 h (*n* = 117)	*p*-Value
History of PONV, yes	191 (17.8)	48 (41.0)	<0.001
Analgesic-related ADR with NV	141 (13.2)	40 (34.2)	<0.001
General anesthesia	97 (9.1)	13 (11.1)	0.576
LIA use	910 (85.0)	96 (82.1)	0.486
Dexamethasone use	307 (28.7)	24 (20.5)	0.079

Values were presented by frequency (percentage) or mean ± standard deviation; *p*-values were calculated by chi-square test for categorical variables and independent two-sample *t*-test for continuous variables, as appropriate; ADR, adverse drug reaction; LIA, Local infiltration analgesia; NV, nausea/vomiting; PCA, patient-controlled analgesia; PONV, postoperative nausea and vomiting.

**Table 3:** Comparison of model performance across three feature sets reflecting incremental clinical information for predicting PCA discontinuation within 24 h.

Feature Set	Model	CV AUC (Mean ± SD)	CV PR-AUC (Mean ± SD)	Test AUC (95% CI)	Test PR-AUC (95% CI)	Brier Score
Step 1	LR	0.485 ± 0.069	0.129 ± 0.049	0.546 (0.383–0.686)	0.189 (0.131–0.335)	0.066
	XGBoost	0.480 ± 0.045	0.097 ± 0.015	0.622 (0.475–0.769)	0.315 (0.280–0.419)	0.074
	RF	0.582 ± 0.098	0.151 ± 0.052	0.664 (0.510–0.817)	0.212 (0.173–0.314)	0.067
Step 2	LR	0.653 ± 0.055	0.232 ± 0.062	0.769 (0.603–0.934)	0.303 (0.211–0.502)	0.066
	XGBoost	0.610 ± 0.064	0.161 ± 0.012	0.720 (0.574–0.868)	0.299 (0.258–0.388)	0.076
	RF	0.693 ± 0.067	0.263 ± 0.055	0.782 (0.608–0.951)	0.340 (0.234–0.509)	0.066
Step 3	LR	0.684 ± 0.064	0.260 ± 0.070	0.753 (0.585–0.922)	0.311 (0.202–0.549)	0.065
	XGBoost	0.593 ± 0.075	0.175 ± 0.031	0.740 (0.583–0.885)	0.318 (0.267–0.444)	0.074
	RF	0.697 ± 0.082	0.275 ± 0.083	0.773 (0.600–0.941)	0.378 (0.273–0.549)	0.066

Cross-validated performance is reported as mean ± standard deviation using five-fold stratified cross-validation in the training set. Ninety-five percent confidence intervals for test-set AUC and PR-AUC values were estimated using bootstrap resampling with 1000 iterations. Brier score was calculated on the independent test set, with lower values indicating better calibration performance; AUC, area under the receiver operating characteristic curve; CV, cross-validated; LR, logistic regression; PCA, patient-controlled analgesia; PR-AUC, area under the precision–recall curve; RF, random forest.

## Data Availability

The data presented in this study are available on request from the corresponding author.

## Supplementary Materials

The following supporting information can be downloaded at: https://www.mdpi.com/article/10.3390/jcm15114282/s1. Table S1: Feature importance, mean absolute SHAP values, and direction of association for the logistic regression model across Step 1, Step 2, and Step 3 feature sets; Table S2: Feature importance, mean absolute SHAP values, and direction of association for the XGBoost model across Step 1, Step 2, and Step 3 feature sets; Table S3: Feature importance, mean absolute SHAP values, and direction of association for the random forest model across Step 1, Step 2, and Step 3 feature sets; Table S4: Inclusion and exclusion criteria; Table S5: Threshold-dependent classification metrics across machine learning models and feature sets evaluated on the independent test set; TRIPODAI_checklist; Figure S1: SHAP summary plot for the random forest model using the Step 3 feature set; Figure S2: Receiver operating characteristic (ROC) and precision–recall (PR) curves of the logistic regression model across stepwise feature sets evaluated on the independent test set; Figure S3: Receiver operating characteristic (ROC) and precision–recall (PR) curves of the XGBoost model across stepwise feature sets evaluated on the independent test set.

## Abbreviations

The following abbreviations are used in this manuscript:

ADR	Adverse drug reaction
AUC	Area under the receiver operating characteristic curve
BMI	Body mass index
CeVD	Cerebrovascular disease
CKD	Chronic kidney disease
CLD	Chronic lung disease
CV	Cross-validation/Cross-validated
CVD	Cardiovascular disease
DCA	Decision curve analysis
DM	Diabetes mellitus
EMR	Electronic medical record
ERAS	Enhanced Recovery After Surgery
HTN	Hypertension
IRB	Institutional Review Board
LIA	Local infiltration analgesia
LR	Logistic regression
ML	Machine learning
NRS	Numeric rating scale
NV	Nausea and vomiting
PCA	Patient-controlled analgesia
PONV	Postoperative nausea and vomiting
PR	Precision–recall
PR-AUC	Area under the precision–recall curve
PROMs	Patient-reported outcome measures
RA-TKA	Robotic-assisted total knee arthroplasty
RF	Random forest
ROC	Receiver operating characteristic
SHAP	SHapley Additive exPlanations
SMOTE	Synthetic minority oversampling technique
TKA	Total knee arthroplasty
TRIPOD + AI	Transparent Reporting of a multivariable prediction model for Individual Prognosis Or Diagnosis + Artificial Intelligence
VAS	Visual analog scale
XGBoost	Extreme gradient boosting
