# Supplementary material for: Machine Learning-Based Prediction of Early Patient-Controlled Analgesia Discontinuation After Total Knee Arthroplasty: A Retrospective Cohort Study
Source: J Clin Med. 2026 Jun 1;15(11):4282. doi: 10.3390/jcm15114282 (PMC13257909; doi:10.3390/jcm15114282)
Supplement: Supplementary file 1 [file jcm-15-04282-s001.zip › jcm-4346951-supplementary.pdf]

Supplementary Materials

Hyperparameter Optimization

Hyperparameter tuning was performed using a structured grid search within the training dataset only. For each machine learning algorithm, candidate hyperparameter combinations were evaluated using stratified five-fold cross-validation, and the configuration yielding the highest mean area under the receiver operating characteristic curve (AUC) was selected. This approach was adopted to optimize predictive performance while minimizing the risk of information leakage.

Once optimal hyperparameters were identified, the same parameter settings were applied consistently across all three hierarchical feature sets (Step 1: baseline patient characteristics and comorbidities; Step 2: Step 1 plus PONV-related susceptibility factors; Step 3: Step 2 plus perioperative management variables). This strategy ensured a fair comparison of model performance across feature sets while avoiding bias introduced by feature-set–specific tuning.

For logistic regression, L2 regularization was applied, and the regularization strength was selected using cross-validation. The maximum number of iterations was increased to ensure model convergence, and no additional hyperparameter tuning was performed beyond regularization settings. Random forest models were trained using 900 trees with a maximum tree depth of 6 and a minimum of 20 samples per leaf node to control model complexity and reduce overfitting. The XGBoost model employed 500 boosting rounds with a learning rate of 0.05, maximum tree depth of 3, subsampling ratio of 0.85, and column subsampling ratio of 0.85.

All optimized hyperparameters were fixed prior to evaluation on the independent test set and were not modified during subsequent analyses.

Supplementary Tables: Overview and Index

The Supplementary Tables provide a comprehensive comparison of feature importance, mean absolute SHAP values, and direction of association across multiple machine learning models and incremental feature sets for predicting patient-controlled analgesia discontinuation within 24 hours after total knee arthroplasty.
Three commonly used predictive modeling approaches—logistic regression, XGBoost, and random forest—were evaluated using three stepwise feature sets that reflect increasing availability of clinical information. Step 1 includes baseline patient characteristics and comorbidities; Step 2 extends Step 1 by incorporating PONV-related susceptibility factors; and Step 3 further adds perioperative management variables.
Feature importance metrics are model-specific and therefore not directly comparable across different algorithms. To facilitate consistent interpretation across models, mean absolute SHAP values are additionally presented as a unified measure of each feature's contribution to model predictions. Direction of association summarizes whether each feature is associated with an increased (+), decreased (−), or no clear (0) risk of patient-controlled analgesia discontinuation within 24 hours, relative to PCA maintenance, based on the sign and consistency of SHAP contributions in the test set.

Index of Supplementary Tables

- **Supplementary Table S1.**
  Feature importance, mean absolute SHAP values, and direction of association for the **logistic regression** model across Step 1, Step 2, and Step 3 feature sets.
- **Supplementary Table S2.**
  Feature importance, mean absolute SHAP values, and direction of association for the **XGBoost** model across Step 1, Step 2, and Step 3 feature sets.
- **Supplementary Table S3.**
  Feature importance, mean absolute SHAP values, and direction of association for the **random forest** model across Step 1, Step 2, and Step 3 feature sets.
- **Supplementary Table S4.**
  Inclusion and exclusion criteria.
- **Supplementary Table S5.**
  Threshold-dependent classification metrics, including sensitivity, specificity, positive predictive value, and negative predictive value, across machine learning models and feature sets.

Supplementary Table S1. Feature importance, mean absolute SHAP values, and direction of association for the logistic regression model across Step 1, 2, and 3 feature sets.

Feature sets		Feature importance			Mean(|SHAP|)			Direction of association		
		Step 1	Step 2	Step 3	Step 1	Step 2	Step 3	Step 1	Step 2	Step 3
Patient characteristics and comorbidities	Age	0.012	0.003	0.001	0.070	0.017	0.006	-	-	+
	BMI	0.037	0.036	0.032	0.142	0.136	0.122	+	+	+
	Female	0.324	0.234	0.307	0.094	0.068	0.089	+	+	+
	Smoking	0.722	0.786	0.797	0.127	0.138	0.140	-	-	-
	HTN	0.035	0.000	0.020	0.017	0.000	0.010	+	-	-
	DM	0.067	0.095	0.092	0.020	0.028	0.028	-	-	-
	CVD	0.183	0.095	0.137	0.041	0.021	0.031	+	+	+
	CeVD	0.268	0.165	0.069	0.032	0.020	0.008	+	+	+
	CLD	0.257	0.303	0.275	0.006	0.007	0.006	-	-	-
	CKD	0.528	0.611	0.618	0.026	0.030	0.030	-	-	-
PONV-related susceptibility factors	History of PONV		1.167	1.223		0.413	0.433		+	+
	Analgesic-related ADR with NV		1.106	1.085		0.246	0.241		+	+
Perioperative management factors	General anesthesia			0.167			0.025			-
	LIA use			0.570			0.148			-
	Dexamethasone use			0.596			0.243			-

Step 1 includes baseline patient characteristics and comorbidities; Step 2 additionally incorporates PONV-related susceptibility factors; and Step 3 further includes perioperative management variables. Feature importance values for logistic regression represent the absolute values of standardized regression coefficients. Mean(|SHAP|) indicates the mean absolute SHAP value for each predictor, reflecting its overall contribution to the prediction of patient-controlled analgesia discontinuation within 24 hours. Direction of association indicates whether higher values (or the presence of a category relative to the reference) are associated with an increased (+), decreased (−), or no clear (0) risk of patient-controlled analgesia discontinuation within 24 hours, based on the sign and consistency of SHAP contributions in the test set. SHAP, SHapley Additive exPlanations; PONV, postoperative nausea and vomiting; BMI, body mass index; HTN, hypertension; DM, diabetes mellitus; CVD, cardiovascular disease; CeVD, cerebrovascular disease; CLD, chronic lung disease; CKD, chronic kidney disease; ADR, adverse drug reaction; NV, nausea/vomiting; LIA, local infiltration analgesia.

Supplementary Table S2. Feature importance, mean absolute SHAP values, and direction of association for the XGBoost model across Step 1, 2, and 3 feature sets.

Feature sets		Feature importance			Mean(|SHAP|)			Direction of association		
		Step 1	Step 2	Step 3	Step 1	Step 2	Step 3	Step 1	Step 2	Step 3
Patient characteristics and comorbidities	Age	0.101	0.068	0.058	0.530	0.574	0.545	-	+	+
	BMI	0.107	0.072	0.062	0.546	0.577	0.561	+	-	+
	Female	0.097	0.061	0.059	0.177	0.130	0.153	+	+	+
	Smoking	0.126	0.085	0.065	0.151	0.163	0.162	-	-	-
	HTN	0.083	0.064	0.041	0.084	0.079	0.084	-	-	-
	DM	0.098	0.065	0.055	0.079	0.140	0.108	-	-	-
	CVD	0.127	0.073	0.062	0.052	0.053	0.071	-	+	+
	CeVD	0.096	0.066	0.057	0.030	0.022	0.023	+	+	-
	CLD	0.077	0.042	0.029	0.005	0.002	0.002	-	-	-
	CKD	0.088	0.060	0.035	0.026	0.012	0.013	-	-	-
PONV-related susceptibility factors	History of PONV		0.190	0.145		0.477	0.532		+	+
	Analgesic-related ADR with NV		0.153	0.126		0.446	0.416		+	+
Perioperative management factors	General anesthesia			0.055			0.040			-
	LIA use			0.083			0.183			-
	Dexamethasone use			0.069			0.236			-

Step 1 includes baseline patient characteristics and comorbidities; Step 2 additionally incorporates PONV-related susceptibility factors; and Step 3 further includes perioperative management variables. For the XGBoost model, feature importance values represent the gain-based importance, which quantifies the average improvement in model accuracy brought by splits using each feature across all boosted trees. Mean(|SHAP|) indicates the mean absolute SHAP value for each predictor, reflecting its overall contribution to the prediction of patient-controlled analgesia discontinuation within 24 hours. Direction of association indicates whether higher values (or the presence of a category relative to the reference) are associated with an increased (+), decreased (−), or no clear (0) risk of patient-controlled analgesia discontinuation within 24 hours, based on the sign and consistency of SHAP contributions in the test set. SHAP, SHapley Additive exPlanations; PONV, postoperative nausea and vomiting; BMI, body mass index; HTN, hypertension; DM, diabetes mellitus; CVD, cardiovascular disease; CeVD, cerebrovascular disease; CLD, chronic lung disease; CKD, chronic kidney disease; ADR, adverse drug reaction; NV, nausea/vomiting; LIA, local infiltration analgesia.

Supplementary Table S3. Feature importance, mean(|SHAP|), and direction of association for the Random forest model across Step 1, 2, and 3 feature sets.

Feature sets		Feature importance			Mean(|SHAP|)			Direction of association		
		Step 1	Step 2	Step 3	Step 1	Step 2	Step 3	Step 1	Step 2	Step 3
Patient characteristics and comorbidities	Age	0.388	0.231	0.200	0.015	0.012	0.010	-	-	-
	BMI	0.433	0.235	0.237	0.017	0.011	0.011	-	+	+
	Female	0.036	0.015	0.014	0.005	0.002	0.002	+	+	+
	Smoking	0.034	0.013	0.014	0.004	0.003	0.002	-	-	-
	HTN	0.040	0.020	0.019	0.002	0.001	0.001	-	-	-
	DM	0.024	0.021	0.017	0.001	0.001	0.002	-	-	-
	CVD	0.025	0.011	0.012	0.002	0.002	0.001	+	+	+
	CeVD	0.019	0.009	0.006	0.001	0.001	0.001	+	+	+
	CLD	0.000	0.000	0.000	0.000	0.000	0.000	0	0	0
	CKD	0.001	0.000	0.000	0.000	0.000	0.000	-	-	-
PONV-related susceptibility factors	History of PONV		0.229	0.214		0.024	0.022		+	+
	Analgesic-related ADR with NV		0.215	0.192		0.019	0.016		+	+
Perioperative management factors	General anesthesia			0.007			0.001			-
	LIA use			0.037			0.005			-
	Dexamethasone use			0.031			0.005			-

Step 1 includes baseline patient characteristics and comorbidities; Step 2 additionally incorporates PONV-related susceptibility factors; and Step 3 further includes perioperative management variables. Feature importance values for the Random Forest model represent the number of tree splits using each predictor across all trees. Mean(|SHAP|) indicates the mean absolute SHAP value for each predictor, reflecting its overall contribution to the prediction of patient-controlled analgesia discontinuation within 24 hours. Direction of association indicates whether higher values (or the presence of a category relative to the reference) are associated with an increased (+), decreased (−), or no clear (0) risk of patient-controlled analgesia discontinuation within 24 hours, based on the sign and consistency of SHAP contributions in the test set. SHAP, SHapley Additive exPlanations; PONV, postoperative nausea and vomiting; BMI, body mass index; HTN, hypertension; DM, diabetes mellitus; CVD, cardiovascular disease; CeVD, cerebrovascular disease; CLD, chronic lung disease; CKD, chronic kidney disease; ADR, adverse drug reaction; NV, nausea/vomiting; LIA, local infiltration analgesia.

Supplementary Table S4. Inclusion and exclusion criteria.

Inclusion Criteria
Adult patients undergoing primary unilateral total knee arthroplasty
Postoperative patient-controlled analgesia (PCA) initiated immediately after surgery
Availability of PCA maintenance or discontinuation status within 24 hours after surgery
Surgery performed between December 1, 2015, and November 30, 2025
Exclusion Criteria
Bilateral total knee arthroplasty
No postoperative PCA application
Unclear PCA discontinuation status within 24 hours
Major intraoperative or immediate postoperative complications precluding PCA evaluation
Intensive care unit admission within 24 hours after surgery
Reoperation within 24 hours after surgery
Death within 24 hours after surgery
Substantial missing data in the outcome variable or key perioperative predictors

Supplementary Table S5. Threshold-dependent classification metrics across machine learning models and feature sets evaluated on the independent test set.

Threshold	Model	Feature Set	Sensitivity	Specificity	PPV	NPV
0.05	Logistic regression	Step 1	0.765	0.199	0.068	0.917
0.05	Logistic regression	Step 2	0.706	0.480	0.094	0.955
0.05	Logistic regression	Step 3	0.706	0.475	0.094	0.955
0.05	Random forest	Step 1	0.824	0.226	0.076	0.943
0.05	Random forest	Step 2	0.647	0.362	0.072	0.930
0.05	Random forest	Step 3	0.706	0.258	0.068	0.919
0.05	XGBoost	Step 1	0.353	0.665	0.075	0.930
0.05	XGBoost	Step 2	0.353	0.688	0.080	0.933
0.05	XGBoost	Step 3	0.412	0.674	0.089	0.937
0.1	Logistic regression	Step 1	0.059	0.910	0.048	0.926
0.1	Logistic regression	Step 2	0.294	0.855	0.135	0.940
0.1	Logistic regression	Step 3	0.353	0.851	0.154	0.945
0.1	Random forest	Step 1	0.118	0.946	0.143	0.933
0.1	Random forest	Step 2	0.353	0.833	0.140	0.944
0.1	Random forest	Step 3	0.294	0.851	0.132	0.940
0.1	XGBoost	Step 1	0.176	0.824	0.071	0.929
0.1	XGBoost	Step 2	0.294	0.833	0.119	0.939
0.1	XGBoost	Step 3	0.294	0.846	0.128	0.940
0.15	Logistic regression	Step 1	0.000	1.000	NA	0.929
0.15	Logistic regression	Step 2	0.294	0.882	0.161	0.942
0.15	Logistic regression	Step 3	0.294	0.882	0.161	0.942
0.15	Random forest	Step 1	0.000	1.000	NA	0.929
0.15	Random forest	Step 2	0.118	0.937	0.125	0.932
0.15	Random forest	Step 3	0.059	0.968	0.125	0.930
0.15	XGBoost	Step 1	0.059	0.896	0.042	0.925
0.15	XGBoost	Step 2	0.176	0.882	0.103	0.933
0.15	XGBoost	Step 3	0.176	0.910	0.130	0.935

Step 1 includes baseline demographic characteristics and comorbidities; Step 2 additionally incorporates PONV-related susceptibility factors; and Step 3 further includes perioperative management variables. Threshold-dependent metrics should be interpreted as exploratory because no single clinically predefined probability threshold was established in this study. PPV was not estimable when no positive predictions were generated at the selected threshold. NPV, negative predictive value; PPV, positive predictive value.

Supplementary Figure Legends

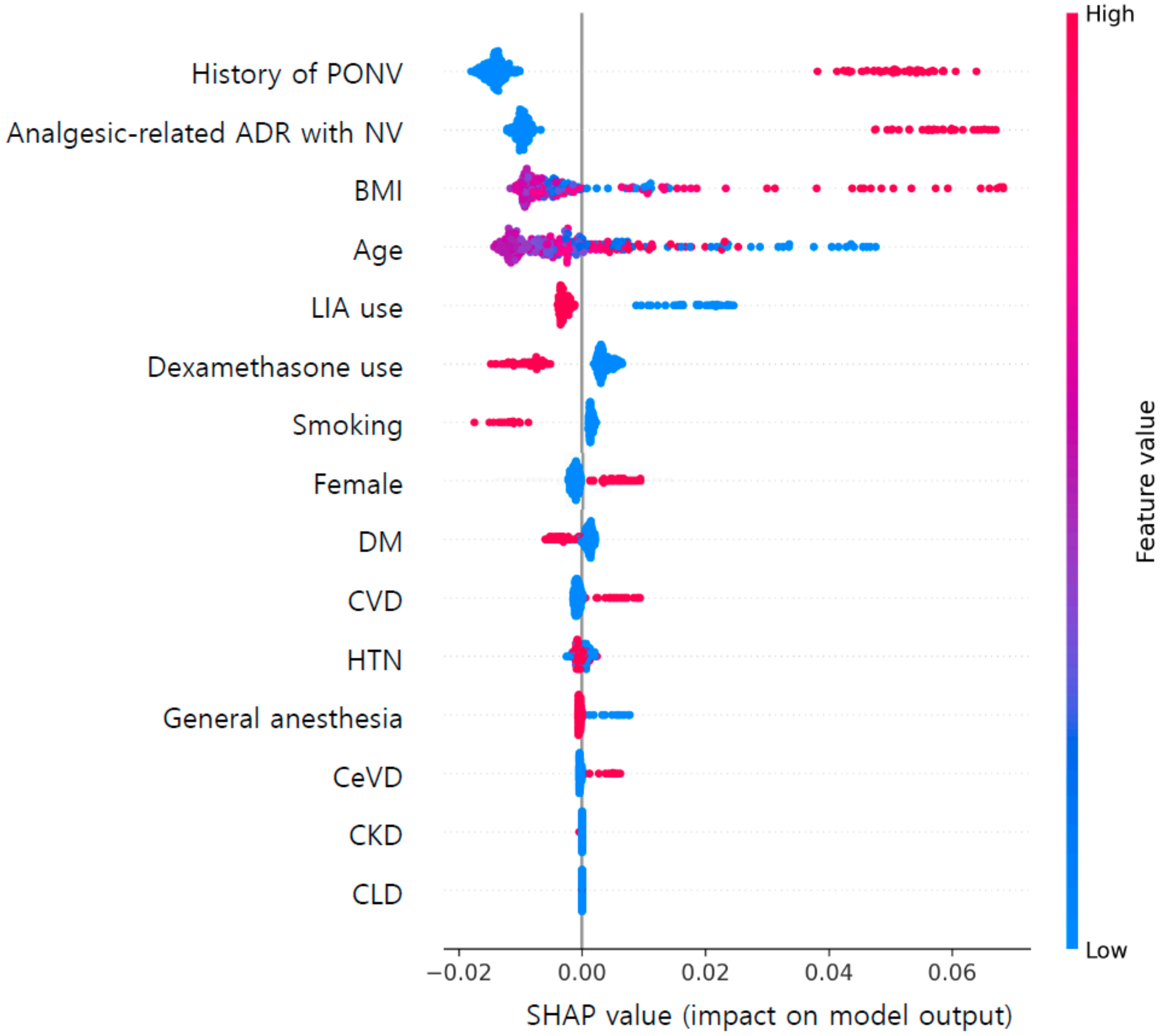

Supplementary Figure S1. SHAP summary plot for the random forest model using the Step 3 feature set. Features are ranked according to mean absolute SHAP values, reflecting their relative contribution to model predictions. ADR, adverse drug reaction; BMI, body mass index; CeVD, cerebrovascular disease; CKD, chronic kidney disease; CLD, chronic lung disease; CVD, cardiovascular disease; DM, diabetes mellitus; HTN, hypertension; LIA, local infiltration analgesia; NV, nausea/vomiting; RF, random forest; SHAP, SHapley Additive exPlanations.

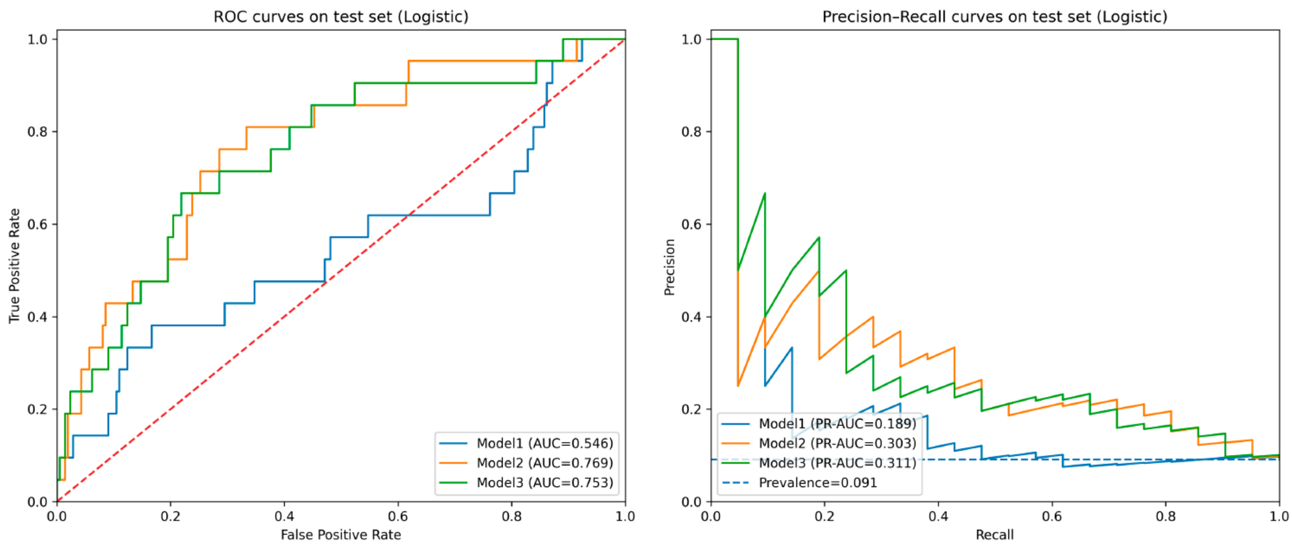

Supplementary Figure S2. Receiver operating characteristic (ROC) and precision–recall (PR) curves of the logistic regression model across stepwise feature sets evaluated on the independent test set. The diagonal dashed line in the ROC panel represents chance-level discrimination, while the horizontal dashed line in the PR panel denotes the event prevalence in the test set. AUC, area under the receiver operating characteristic curve; LR, logistic regression; PR, precision–recall; PR-AUC, area under the precision–recall curve; ROC, receiver operating characteristic.

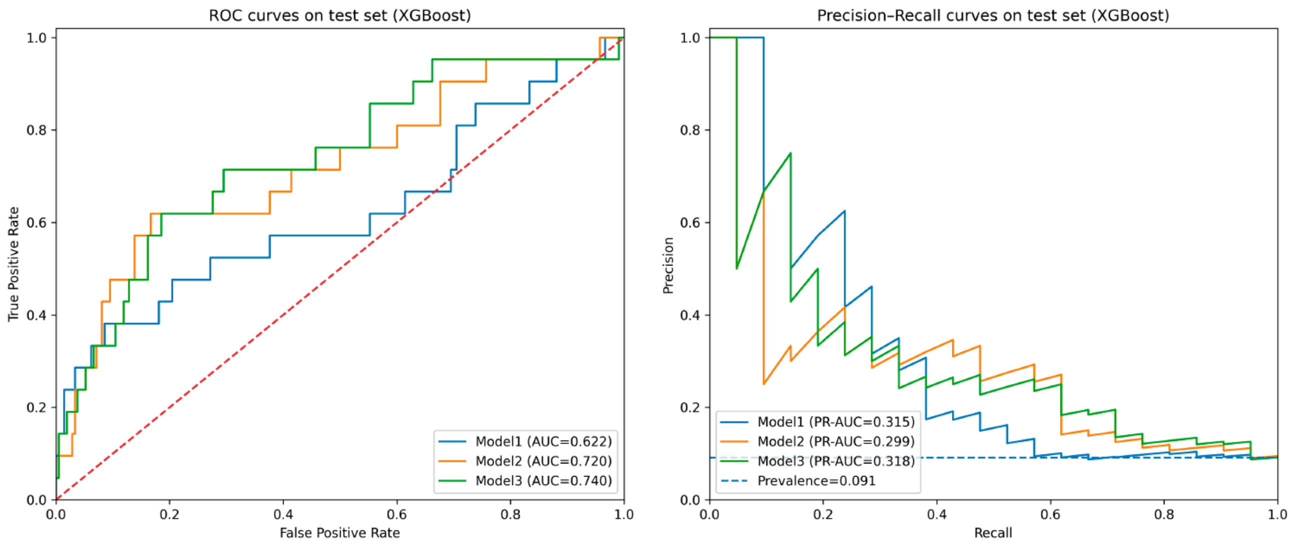

Supplementary Figure S3. Receiver operating characteristic (ROC) and precision–recall (PR) curves of the XGBoost model across stepwise feature sets evaluated on the independent test set. The diagonal dashed line in the ROC panel represents chance-level discrimination, while the horizontal dashed line in the PR panel denotes the event prevalence in the test set. AUC, area under the receiver operating characteristic curve; PR, precision–recall; PR-AUC, area under the precision–recall curve; ROC, receiver operating characteristic.
